# A Systematic Review of the Effects of Exercise on Hormones in Women with Polycystic Ovary Syndrome

**DOI:** 10.3390/jfmk5020035

**Published:** 2020-05-31

**Authors:** Grei Shele, Jessica Genkil, Diana Speelman

**Affiliations:** Department of Biochemistry, Lake Erie College of Osteopathic Medicine, Erie, PA 16509, USA; gshele34526@med.lecom.edu (G.S.); jgenkil35353@med.lecom.edu (J.G.)

**Keywords:** physical exercise, PCOS, obesity, androgens, hyperandrogenism, insulin resistance, adipokines

## Abstract

Background: Polycystic ovary syndrome (PCOS) is a common endocrine disorder that is characterized by menstrual irregularity and elevated serum androgens, and is often accompanied by insulin resistance. The etiology of PCOS is unknown. Lifestyle interventions and weight loss, where appropriate, remain first-line treatments for women with PCOS. Regular physical activity is recommended for women with PCOS to maintain a healthy weight and cardiovascular fitness. Purpose: To review the evidence for the impact of various exercise interventions on hormone levels in women with PCOS. Methods: A systematic review of original studies indexed in PubMed that utilized an exercise intervention in women with PCOS and reported hormone values pre- and post-intervention. Studies in which the effects of the exercise intervention could be determined were included. Results: Vigorous aerobic exercise improves insulin measures in women with PCOS. Resistance or strength training may improve androgen levels, though additional studies are warranted. Studies with yoga are limited but suggest improvements in androgens. Limited information is available on the impact of exercise on adipokines and anti-Müllerian hormone, warranting further investigation. Conclusions: Recommended guidelines for women with PCOS include vigorous aerobic exercise and resistance training to improve measures of insulin sensitivity and androgen levels.

## 1. Introduction

### 1.1. Polycystic Ovary Syndrome

Polycystic ovary syndrome (PCOS) is the most common hormone disorder in women of reproductive age, affecting 5–15% of women as early as the second decade of life [1,2,3]. As the exact causes of the disorder remain unknown, it is diagnosed based upon consensus criteria and the exclusion of other endocrine disorders (Table 1), with the Rotterdam criteria most commonly used for diagnosis [4,5,6,7]. PCOS is characterized by hyperandrogenism and oligo-ovulation or anovulation, resulting in acne, hirsutism, male-pattern hair loss, irregular menstrual cycles, and subfertility [1,8]. In addition to the endocrine and reproductive effects, PCOS impacts cardiometabolic and psychological health across the lifespan [3,9,10,11,12,13]. Women with the disorder are more likely to develop obesity, type 2 diabetes, cardiovascular disease (CVD), non-alcoholic fatty liver disease (NAFLD), and have a greater risk of developing anxiety, depression, and mood disorders [10,11,14,15,16,17,18,19,20,21,22,23,24,25,26,27,28,29,30,31,32,33].

In women with PCOS, the ovaries are stimulated to produce excessive amounts of androgens; this may be through hyperstimulation by luteinizing hormone (LH) or the intrinsic dysregulation of steroidogenesis [1,34,35]. In addition to contributing to the development of many features of the disorder, hyperandrogenism promotes insulin resistance and hyperinsulinemia, which can further exacerbate hyperandrogenemia as well as lead to weight gain and obesity [36,37,38,39]. The mechanisms by which androgens specifically affect insulin dynamics are unknown. However, insulin resistance in women with PCOS has been attributed to increased insulin receptor and insulin receptor substrate 1 (IRS1) serine phosphorylation in muscle [40], with impaired insulin signaling that affects the metabolic but not the mitogenic pathway [41,42,43,44]. Insulin resistance, hyperinsulinemia, and overweight or obesity are common in women with PCOS and worsen the manifestation of the disorder, in part by further stimulating steroidogenesis and androgen production [13,45,46,47,48,49]. The reciprocal relationship between androgens and insulin, combined with the impact of each on the presentation of the features of PCOS and the unknown etiology of the disorder, make PCOS a challenging endocrine disorder to treat [50,51,52,53,54,55]. In addition to elevated levels of androgens and insulin, adipose-derived hormones are reported to be dysregulated in PCOS and may further contribute to metabolic dysfunction in women with the disorder [56,57,58,59].

### 1.2. Rationale for Systematic Review

#### 1.2.1. Hormones Dysregulated in PCOS

PCOS is an endocrine disorder involving elevated androgen levels and often insulin resistance and hyperinsulinemia. Most often, the androgen used to assess hyperandrogenemia is free testosterone (fT), although total testosterone (TT), sex hormone binding globulin (SHBG), the free androgen index (FAI), dehydroepiandrosterone (DHEA), DHEA sulfate (DHEA-S), and androstenedione (A4) may also be altered [6]. The pituitary-derived luteinizing hormone (LH) is also commonly elevated in women with PCOS and stimulates ovarian overproduction of androgens [46,60,61]. Anti-Müllerian hormone (AMH) is produced by the ovaries and is an indicator of ovarian reserve, which declines as a function of age [62]. Serum AMH levels are higher in women with PCOS, particularly those with a more severe phenotypic presentation [63]. AMH has diagnostic value and may help predict the response to pharmacologic ovulation induction, but does not appear to contribute to PCOS pathogenesis directly [64,65,66,67].

In addition to these hormones, altered levels of several adipokines have been reported in women with PCOS. Adiponectin, an adipose-derived hormone involved in insulin sensitization and fuel disposal from the blood, is lower in women with PCOS and as a result may contribute to insulin resistance [56,57,58,59,68,69]. Similarly, plasma visfatin is also increased in women with PCOS and may promote insulin resistance [70,71,72,73,74,75]. Resistin is an adipocytokine that promotes inflammation and may contribute to PCOS pathogenesis independent of insulin resistance [76,77,78]. Leptin, an adipokine involved in the regulation of long-term food intake and energy expenditure, is elevated in PCOS and is associated with increased cardiometabolic risk [58,79,80]. These adipokines may also serve as biomarkers in PCOS [81].

#### 1.2.2. Role of Physical Activity in Managing PCOS Symptoms

One of the first lines of advice for managing PCOS symptoms is weight loss, where appropriate, coupled with a healthy, well-balanced diet [50,53,82]. In addition, women with PCOS are given the general advice to be physically active [55,83]. Studies have examined different types of exercise and exercise regimens in mitigating PCOS features and improving health and outcomes in women with the disorder. In this review, we evaluate the findings of the investigations that reported hormone values.

### 1.3. Objectives of Review

The goals of this systematic review are to evaluate the study design and results of investigations of exercise on hormones in women with PCOS, to use this information to guide therapeutic recommendations for women with the disorder, and to highlight the need for future studies. We summarize the demographics of participants in each study, types of exercise interventions utilized, study design, hormone outcomes measured, and limitations of each study. Finally, we use this information to synthesize recommendations for guidelines for physical activity in women with PCOS.

## 2. Materials and Methods

### 2.1. Literature Search

**Figure 1:**
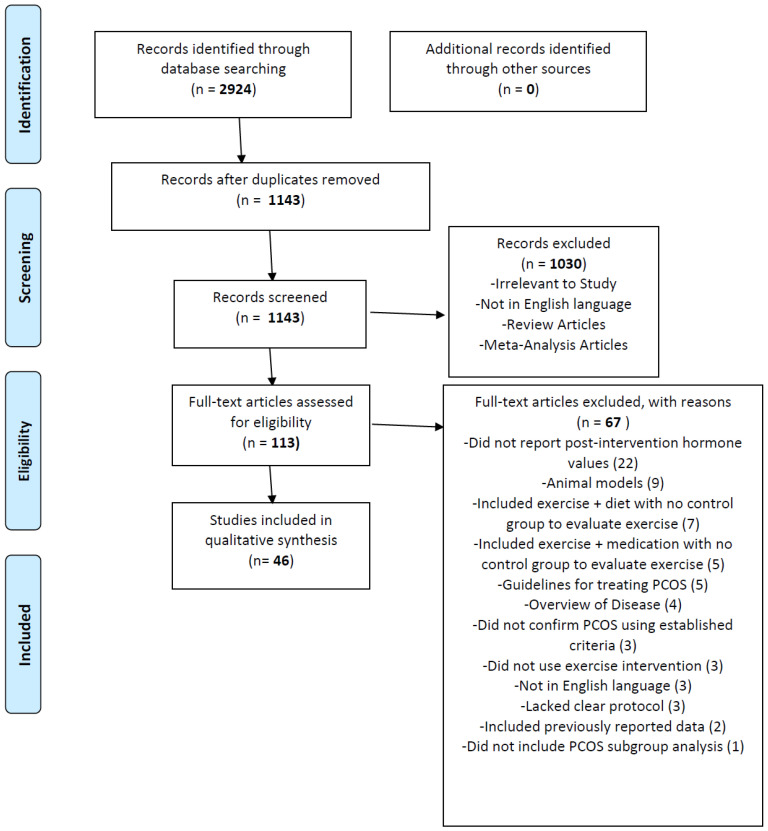
PRISMA flow diagram showing the literature search, eligibility, and selection for the inclusion of studies in the systematic review.

This systematic review was carried out in accordance with the Preferred Reporting Items for Systematic Reviews and Meta-Analyses (PRISMA), which is summarized in Figure 1. A comprehensive search of the literature was conducted by using the PubMed database on 2 March 2020 to 14 April 2020. The search strategy included an exercise term: “exercise” or “aerobic” or “physical activity” or “resistance training” or “high intensity interval” or “yoga” or “strength training” or “endurance training” or “HIIT”, paired with a PCOS term: “PCOS” or “polycystic ovary” or “polycystic ovarian”. In total there were 27 separate searches. A list of all articles returned by the searches was made and all duplicates were removed.

### 2.2. Eligibility Criteria, Article Screening, and Exclusion Criteria

Three study investigators independently reviewed the titles and abstracts retrieved from the initial literature search based on the relevance to the systematic review goals. Review articles and meta-analyses were removed, leaving original research articles. Full text articles were then screened for those that included an exercise or physical activity intervention in a population of women with PCOS and reported at least one hormone measurement. Articles excluded were studies that did not report any post-intervention hormone values [23], used an animal model [9], included exercise only in combination with diet with no control group to evaluate the effects of exercise [8], included exercise only in combination with medication with no control group to evaluate the effects of exercise [5], provided only guidelines for treating women with PCOS [5] or an overview of the disease [4], did not confirm a PCOS diagnosis using established criteria [3], did not use an exercise intervention [3], were not in English [3], lacked a clear protocol [3], included previously reported data [2], or included subjects both with PCOS and without and did not include a PCOS subgroup analysis [1].

### 2.3. Classification of Articles and Qualitative Synthesis

Articles were classified according to the type of exercise intervention for the purpose of synthesis of the information: aerobic exercise, combination exercises or high-intensity interval training or exercise in conjunction with dietary modification, progressive resistance training or strength training, yoga, or single bout exercise. Studies included randomized controlled trials, non-randomized controlled trials, case–control studies, and single-arm prospective studies with pre- and post-intervention measurements.

### 2.4. Data Collection

Full text articles meeting the eligibility criteria for a qualitative synthesis review were reviewed and data were extracted independently by three investigators. Data were extracted and include participant demographic information, criteria used to confirm PCOS status, procedure for participant assignment to a group, number of participants in the final data analysis, protocol for exercise intervention (type, frequency, duration), and hormones reported pre- and post-intervention. Principle summary measures used were the difference in means and a statistical analysis reported with data in the primary investigations. The extracted data are summarized in Tables 2–5.

### 2.5. Risk of Bias

Risk of bias in individual studies was classified as selection bias (e.g., non-randomized trials), performance bias (e.g., insufficient control of variables outside of the study protocol), attrition bias, and reporting bias (e.g., not reporting data with non-significant differences). These biases were used in evaluating the strength of the data reported and in synthesizing recommended guidelines for patients. All articles, regardless of the results or conclusion of the study, were included in the qualitative synthesis review in order to avoid selection bias across studies.

## 3. Results

### 3.1. Aerobic Exercise

Studies of aerobic exercise interventions in women with PCOS that included hormone outcomes measured are summarized in Table 2. Randomized controlled trials (RCTs) with aerobic exercise interventions in studies of women with PCOS included prescribed exercise on a motorized treadmill or cycling under the supervision of medical, athletic, or research personnel. In a three-month study, cycling for 30 min three days per week at 60–70% VO_2_max resulted in decreased fasting insulin (FI) levels, with no significant changes in the sex hormones, LH, or sex hormone binding globulin (SHBG) [84,85]. Similar findings were reported following a 24-week structured program with the same cycling parameters, and the improved FI was not maintained in a 12-week program followed by 12-weeks of detraining [86]. Cycling more frequently and for a longer duration during a 12-week period, for 60 min five days per week at 65% VO_2_max, did not yield changes to FI or the homeostatic model assessment of insulin resistance (HOMA-IR), but did result in improvement in insulin sensitivity (IS) and the glucose infusion rate (GIR) during a hyperinsulinemic-euglycemic clamp, as well as a decrease in insulin C-peptide [87]. A six-month study that involved cycling for 45 min three days per week at 60–70% VO_2_max yielded a decrease in FI and HOMA-IR, as well as an increase in GIR during a hyperinsulinemic-euglycemic clamp, with no changes reported in sex hormones, LH, or SHBG [88].

With respect to studies involving walking or jogging, a 16-week progressive aerobic training program supervised on an indoor track for 50 min three days per week did not yield changes in FI or HOMA-IR [89]. However, a 16-week RCT with continuous aerobic training (CAT) or intermittent aerobic training (IAT) on a treadmill resulted in decreased total testosterone (TT) in both exercise groups, and a lower free androgen index (FAI) in the IAT group, with no changes in SHBG, A4, E2, LH, or FSH in either group [90,91,92,93].

Some studies utilized individualized aerobic exercise training programs, in which the prescribed regimen was dependent upon a specified exercise energy expenditure calculation or individual progress as assessed by physiologic measurements. A 16–24-week individualized program adapted from the STRRIDE (Studies of Targeted Risk Reduction Interventions through Defined Exercise) study [94,95] that used a calculated 14 kcal/kg/wk exercise dose yielded a trend toward improved insulin response during an intravenous glucose tolerance test (IVGTT) with a minimal model analysis, although there were no statistically significant changes in FI, 2-h insulin, HOMA-IR, or insulin sensitivity (IS) [96]. A 16-week study in which participants self-selected into the aerobic exercise arm, involving walking for 30–45 min for at least three days per week, did not find any changes to the sex hormones, FI, HOMA-IR, SHBG, LH, adiponectin (APN), or anti-Müllerian hormone (AMH) [97,98,99]. However, in an expanded study that included the 16-week walking intervention plus a follow-up at 32 weeks after study commencement, reductions in free testosterone (fT) and DHEA-S were found at 16 and 32 weeks, and reduced estrone sulfate and estradiol (E2) at 16 weeks [100]. In a non-randomized trial in which participants self-selected to either the no exercise group or the aerobic exercise group, a 24-week structured cycling program of 30–40 min at 60–70% VO_2_max found improvements in ovulatory participants for testosterone (T), FAI, FI, HOMA-IR, glucose-to-insulin ratio, and SHBG at both 12 and 24 weeks [101].

Much of the research on aerobic exercise interventions comes from case–control studies of women with PCOS and age—and BMI-matched women without the disorder. Studies that utilized a 12-week intensified aerobic exercise training on a treadmill for 1 h three days per week resulted in improved GIR [102,103,104], lower FI [103,104] and HOMA-IR [103], and lower AMH [102], with no changes in T, FAI, or SHBG [102,103,104]. A similar aerobic exercise training program using alternating moderate and vigorous intensity on a treadmill for 1 h three days per week also improved GIR, as well as FI [105]. For participants that completed the training program but did not lose ≥5% body fat, an increase in the ratio of GIR to fat free mass (FFM) that corresponded to fasting blood glucose was found, without significant changes in GIR, FI, or HOMA-IR [106]. Longer training programs of 16 weeks on a treadmill at 55% VO_2_max five days per week improved measures of insulin sensitivity, and resulted in a 34% increase in glucose uptake during a hyperinsulinemic-euglycemic clamp with no significant change in insulin, TT, FAI, or SHBG [107,108]. A follow-up report indicated a decrease in the ratio of leptin to high molecular weight (HMW) APN, trends toward lower leptin and DHEA-S, with no change in total APN, HMW APN, or HOMA-IR [109]. However, a study of similar size that utilized a 16-week program of 30 min of moderate intensity aerobic activity at 30% of heart rate reserve (HRR) for three days per week and progressing up to 45 min at 60% HRR for five days per week did not find significant changes in sex hormones, SHBG, FI, HOMA-IR, or LH [110,111]. A shorter, 8-week case–control study using a supervised treadmill exercise at 60% VO_2_max for 1 h three days per week reported improved GDR during a hyperinsulinemic-euglycemic clamp and lipid-induced insulin resistance with no change in TT, FAI, SHBG, or FI [112]. A similar intervention found no significant change in HOMA-IR [113].

A few single-arm intervention studies have also investigated hormone values pre- and post-intervention. In a six-month study involving brisk walking for 20–60 min at least three days per week, insulin and FAI, as well as thyroxine, remained unchanged [114]. A more structured 16-week individualized aerobic training program on a treadmill, at 55% VO_2_max for five days per week, improved GDR and a measure of atrial natriuretic peptide (ANP, −log[EC50]), although TT, FAI, FI, and ANP did not significantly change [107].

Limitations of these studies include selection bias for non-randomized trials, small sample sizes, participant noncompliance with the intervention, reliance on participant self-reported data, lack of monitoring to assess exercise intensity, and high attrition rates.

Taken together, some aerobic exercise training programs, particularly those that involve more vigorous activity and/or more frequent weekly exercise or sessions of longer duration, may improve measures of insulin and insulin responsiveness. The sex hormones, SHBG, and LH appear to be largely unaffected by aerobic exercise, with a few exceptions [90,91,100,101]. Only a couple of studies to date have examined adipokines in women with PCOS after an aerobic exercise intervention, and these show no change in APN [98,109], although one study did find a decrease in the ratio of leptin to HMW APN and a trend toward lower leptin [109]. Two studies have looked at AMH, with one that found no significant change [99] and the other that reported a decrease following the intervention; this may be due to differences in aerobic exercise protocol or participant demographics, and warrants further investigation of specific exercise protocols on AMH levels [102]. Such studies should include measures of menstrual cycle regularity and ovulation, as well as LH and FSH, as a decrease in AMH could be indicative of more regular ovulation and/or the appropriate maturation of follicles.

Further research is warranted to determine if sustained aerobic exercise over a long-term period, or more intense aerobic exercise activity, are able to reduce androgen levels or increase SHBG levels. In addition, the lack of information on the effects of aerobic exercise on adipokines warrants similar investigations.

**Table 2:** Studies of aerobic exercise interventions on hormones in women with PCOS.

Study	Participants	Exercise Intervention	Hormonal Changes	Unchanged
RCT, aerobic ex or no ex [84,85]	90, 124 PCOS (~half/group); mean 22 y; BMI mean 29; Rotterdam criteria	3-month structured ex. training or non-ex. CG; 3 d/wk; 30 min cycling at 60–70% VO_2_max; supervised by medical personnel; continuous electrocardio monitoring	↓FI, AUC_in_	FSH, LH, PRL E2, P, 17-OHP, T, FAI, A4, DHEA-S SHBG
RCT, aerobic ex and detraining [86]	64 PCOS (32/group); BMI mean 29; Rotterdam criteria	24-week structured ex. training or 12-week ex. training plus 12-week detraining; 3 d/wk; 30–40 min cycling at 60–70% VO_2_max; supervised by medical personnel; continuous electrocardio monitoring	↓FI, AUC_ins_ (24 wk ex.)	FSH, LH, PRL E2, P, 17-OHP, T, FAI, A4, DHEA-S SHBG
RCT with aerobic ex. [87] Trials NCT02105428 and NCT01477164	25 PCOS with OB, IR; Rotterdam criteria	12-week aerobic exercise or sedentary control; 5 d/wk; 60 min cycling at 65% VO_2_max	↑GIR ↑IS ↓C-peptide	E2, P FI, HOMA-IR
RCT w/3 arms (ex, OC, or vitamins) [88] Trial NCT00593294	150 PCOS (50/group); 18–40 y; BMI 18–30; NIH criteria	6-month aerobic ex; 3 d/wk for 45 min at 60–70% VO_2_max; cycling; supervised by medical personnel; HRM	↓FI, HOMA-IR (ex) ↑GIR (ex)	FSH, LH, TSH, PRL E2, P, 17-OHP, T, A4, FAI, SHBG DHEA-S
RCT w/2 parallel arms, ex or no ex [89]	27 PCOS, sedentary; 18–34 y; BMI 25–39.9; Rotterdam criteria	16-week progressive aerobic exercise; 3 d/wk for 50 min; indoor track using HRM to regulate walk/jog pace; supervised	N/A (hormones)	FI, HOMA-IR
RCT w/3 parallel arms [90,91,92,93] Trial ISRCTN10416750	69 PCOS (~23/group); 18–39 y, BMI 18–39.9, sedentary; Rotterdam criteria	16-week CAT, IAT, or CG; 3 d/wk; treadmill; HRM for intensity, personal trainer for compliance	↓TT (CAT, IAT groups) ↓FAI (IAT group)	A4, E2 LH, FSH SHBG
RCT, ex vs no ex [96]	20 PCOS; 18–50 y; median BMI 37.9 ex. group; Rotterdam criteria	16–24-week aerobic ex. individualized program, adapted from STRRIDE study, 14 kcal/kg/wk to determine duration w/recheck half-way; supervised and HRM for compliance	AUC_ins_ trended lower	FI, HOMA-IR, 2 h Ins, IS
RCT w/3 parallel arms (electroacupuncture, ex, untreated) [97,98,99] Trial NCT00484705	20 PCOS (5 ex group); BMI 26.8 ± 4.8; Rotterdam criteria 74 PCOS (30 ex group, 2012, 2015 study)	16-week aerobic ex. at self-selected pace; at least 3 d/wk; 30–45 min at faster than normal walking pace; weekly phone call to advise how to increase ex.	N/A Note: Low n (5) in 2009 study	LH, FSH, TT, fT, FAI, DHEA-S SHBG, T4, TSH, IGF-1 FI, HOMA-IR APN (2012 study) AMH (2015 study)
RCT w/3 parallel arms (electroacupuncture, ex, untreated) [100] Trial NCT00484705	74 PCOS (30 ex. group); 30.2 ± 4.7 y; BMI 27.7 ± 6.4; Rotterdam criteria	16-week aerobic ex., self-selected pace; at least 3 d/wk; at least 30 min at faster than normal walking pace; weekly call to advise increase of ex.; 16-wk follow-up (32 wk)	↓fT, DHEA-S (16, 32 wk) ↓E1-S, E2 (16 wk)	T, DHT, DHEA LH, FSH, SHBG
Non-randomized, aerobic ex vs diet [101] Trial NCT00473538	40 PCOS (20 ex. group), self-selected group; 26.8 ± 5.1 y; BMI 33.1 ± 1.3; Rotterdam criteria	24-week structured ex. training; 30–40 min cycling at 60–70% VO_2_max 3 d/wk; supervised by medical personnel; continuous electrocardio monitoring	↓T, FAI (ov., 12, 24 wk) ↓FI, HOMA-IR (ov., 12, 24 wk) ↑glucose:ins (ov., 12, 24 wk) ↑SHBG (ov., 12, 24 wk)	FSH, LH, TSH, PRL E2, P, A4, DHEA-S
Prospective, case-control [102] Trial ISRCTN84763265	7 PCOS, 8 non-PCOS; BMI > 25 NIH criteria	12-week intensified endurance ex training program; 3 d/wk; 1 h; treadmill, alternating intensity (moderate and high)	↑GIR (both groups) ↓AMH (PCOS)	T, FAI, SHBG
Prospective, intensified aerobic ex., case-control [103] Trial ISRCTN84763265	20 PCOS, 14 non-PCOS; 20-40 y, BMI > 27; NIH criteria	12-week intensified aerobic ex.; 3 h/wk; treadmill; supervised by ex. physiologist	↑GIR ↓HOMA-IR, FI	T, FAI, SHBG
Prospective, case-control, aerobic ex [104] Trial ISRCTN84763265	8 PCOS, 8 non-PCOS from subset of earlier study; Rotterdam criteria	12-week individualized, progressive aerobic ex on motorized treadmill; 3 d/wk for 1h; alternate mod. or high intensity; supervised	↓FI ↑GIR (both)	HOMA-IR T, FAI, SHBG
Prospective, case-control [105] Trial ISRCTN84763265	16 PCOS, 13 non-PCOS; BMI > 27; NIH criteria	12-week supervised aerobic ex; treadmill, alternating intensity (moderate and vigorous); 3 d/wk; 1 h; HRM	↓FI ↑GIR	SHBG, T, FAI
Prospective, aerobic ex, case-control [106] Trial ISRCTN84763265	9 PCOS, 7 non-PCOS; BMI > 25, inactive; post-study, categorized as responders (lost ≥ 5% body fat) or nonresponders; Rotterdam criteria	12-week supervised aerobic exercise; 3 d/wk, 45–60 min; treadmill, alternating moderate (20–60 min walk/jog at 75–80% HR_max_) or high (6–8 5 min intervals at 95–100% HR, 2 min rest) intensity; HRM for intensity; compliance monitored by HRM, session sign-ins & duration	↑GIR/FFM (PCOS nonresponders), positively correlated with FBG	GIR, HOMA-IR, FI
Single-arm, aerobic ex [107]	8 HA PCOS; 25 ± 1 y; BMI 32 ± 1.6; controls for baseline comparison only; NIH criteria	16-week supervised and individualized aerobic exercise; 5 d/wk; treadmill at 55% VO_2_max; HRM for ExEE	↑GDR ↑ANP pD_2_ (−log[EC_50_])	TT, FAI, SHBG FI ANP
Prospective, individualized aerobic ex., case-control [108] Trial NCT01150539	8 PCOS, 7 non-PCOS; BMI ≥ 25; NIH criteria	16-week individualized aerobic ex. program; 5 d/wk; treadmill at 55% VO_2_max; supervised	Improved IS (↑glucose, FFA uptake) 34% ↑ glucose uptake	Ins, TT, FAI SHBG
Prospective, aerobic ex., case-control [109] Trial NCT01150539	8 PCOS, 8 non-PCOS; Rotterdam criteria	16-week supervised aerobic ex; 5 d/wk; treadmill at 55% VO_2_max	↓Leptin/HMW APN ↑GDR Trend: ↓leptin, ↑DHEA-S	APN, HMW APN T, FAI, SHBG HOMA-IR
Prospective, case-control or self-selected ex or no ex [110,111]	11 PCOS, 6 non-PCOS; 29 ± 7 y; BMI 34 ± 5; Rotterdam criteria 17 PCOS, self-selected to ex (10) or no ex (7) group; mean 28 y; mean BMI 33; Rotterdam criteria	16-week supervised moderate-intensity aerobic ex on treadmill; 3 d/wk for 30 min at 30% HRR progressing up to 5 d/wk for 45 min at 60% HRR	N/A (hormones) Note: Low n (6–7)	FSH, LH P, E2, T, FAI, SHBG FI, HOMA-IR
Prospective, aerobic ex, case-control [112]	12 PCOS, 10 non-PCOS; Rotterdam criteria	8-week supervised aerobic exercise; 3 d/wk for 1 h; treadmill at 60% VO_2_max	↓GDR Decreased lipid-induced IR	TT, FAI, SHBG FI
Prospective, case-control, aerobic ex [113] Trial ISRCTN42448814	12 PCOS 10 non-PCOS; mean 28.3 y (PCOS); mean BMI 29.4 (PCOS); Rotterdam criteria	8-week supervised aerobic ex; 3 d/wk for 1h; treadmill at 60% VO_2_max	N/A (hormones)	HOMA-IR
Single-arm, aerobic ex [114]	21 PCOS (12 compliant, 9 non); 30.6 ± 6.6 y; BMI 35.5 ± 7.6 NIH criteria	6-month self-selected brisk walking; at least 3 d/wk; 20–60 min; frequency and duration targets increased fortnightly; self-reported diary submitted monthly	N/A (hormones)	Ins FAI T_4_

All studies are longitudinal. Unless otherwise noted, changes are described for PCOS exercise group(s) pre- vs. post-intervention. Abbreviations: A4, androstenedione; AMH, anti-mullerian hormone; ANP, atrial natriuretic peptide; APN, adiponectin; AUC, area under curve; BMI, body mass index; bpm, beats per minute; CAT, continuous aerobic training; CG, control group; d, days; DHEA-S, dehydroepiandrosterone sulfate; E1-S, estrone; E2, estradiol; ex, exercise; ExEE, exercise energy expenditure; FAI, free androgen index; FBG, fasting blood glucose; FFM, fat free mass; FI, fasting insulin; FSH, follicle stimulating hormone; fT, free testosterone; GDR, glucose disposal rate; GIR, glucose infusion rate; HMW, high molecular weight; HOMA-IR, homeostatic model assessment of insulin resistance; HR, heart rate; HRR, heart rate reserve (HRmax–HRrest); HRM, heart rate monitor; IAT, intermittent aerobic training; Ins, insulin; IR, insulin resistance; IS, insulin sensitivity; LH, luteinizing hormone; N/A, not applicable; OB, overweight or obese; OC, oral contraceptive; ov, ovulatory; P, progesterone; PA, physical activity; PRL, prolactin; PRT, progressive resistance training; RCT, randomized controlled trial; SHBG, sex hormone binding globulin; T, testosterone; T4, thyroxine; TSH, thyroid stimulating hormone; TT, total testosterone; wk, week(s); y, years.

### 3.2. High Intensity Interval Training and Combination Exercise Interventions

Studies using high-intensity interval training (HIIT), or exercise in combination with other interventions—more than one type of exercise, exercise plus diet (lifestyle), or exercise plus pharmacologic agent intervention—are summarized in Table 3. HIIT involves several minutes of high-intensity exercise (e.g., ≥90% VO_2_max or maximum heart rate) interspersed with short periods of low-intensity activity or rest, for multiple cycles or repetitions. Four studies using HIIT as an intervention in women with PCOS have indicated changes in several hormones. A case–control study using a 12-week intensified aerobic exercise program with HIIT on a treadmill for an additional eight weeks found improved GIR, but no change in pigment the epithelium-derived factor (the primary outcome measure) [115]. An RCT with a 10-week HIIT intervention resulted in lower FI, HOMA-IR, and DHEA-S, with no change in T, APN, or leptin [116]. A non-randomized prospective study comparing HIIT with moderate-intensity continuous training (MICT) using a treadmill found lower FI and increased APN in the HIIT group participants post-intervention, with no changes in leptin or vaspin [117]. In an RCT comparing 12 weeks of aquatic HIIT in conjunction with metformin versus metformin alone, improvements were seen in fT, TT, FAI, DHEA-S, HOMA-IR, and SHBG; a decrease in LH and an increase in FSH were also reported [118].

Some groups examined aerobic exercise in combination with resistance training, with or without dietary counseling or planning. An RCT comparing dietary modification or diet and combination aerobic exercise and strength training (ST) found a decrease in FI in both the diet and diet plus exercise groups, with no change in the LH to FSH ratio, T, FAI, or SHBG [119]. A 20-week RCT with three parallel arms comparing diet to diet plus aerobic exercise to diet plus aerobic exercise and progressive resistance training (PRT) found improvements in FI and HOMA-IR in both exercise groups at 10 weeks, and lower T and higher SHBG in both exercise groups at 10 and 20 weeks. All groups showed improvement in FI and HOMA-IR, as well as FAI, at 20 weeks [120]. A four-month RCT comparing diet to exercise to diet and exercise utilized moderate aerobic activity and ST, and found no significant changes in T, fT, SHBG, LH, FSH, AMH, FI, HOMA-IR, or IGF-1 [121,122]. Improvements in FI and HOMA-IR, but not in fT, TT, E2, LH, or FSH, were observed in an eight-week RCT comparing aerobic and resistance exercises to no exercise three days per week [123]. In an RCT comparing marching in place exercise to exercise plus metformin over a six-month study period with three 30 min sessions per week, T was unchanged [124].

Limitations of these studies include selection bias for non-randomized trials, small sample sizes, participant noncompliance with the intervention, reliance on participant self-reported data, and high attrition rates.

Studies of HIIT are few but indicate that this type of exercise may provide some benefit in terms of FI and improved HOMA-IR, with potential for improvement in other hormones as well. Larger studies are needed to assess whether these improvements hold, and further investigation is warranted to determine which specific HIIT protocols provide the most benefit. The 12-week intervention with aquatic HIIT improved fT and TT, in addition to FAI, DHEA-S, LH, FSH, SHBG, and HOMA-IR [118]. Although this type of exercise may not be readily accessible to many, it would be worthwhile to investigate more feasible types of HIIT that could potentially mimic this form of exercise and provide similar benefits.

Lifestyle interventions, whether they include aerobic or combined exercise, appear to improve numerous hormones, particularly with more vigorous aerobic activity and in combination with dietary and physical activity counseling. Such benefits may have the greatest impact in obese women with PCOS, suggesting that weight loss may play a role in the hormonal improvements. This would be consistent with improved fertility outcomes with weight loss in women with PCOS [125].

### 3.3. Progressive Resistance Training or Strength Training

Few studies have examined PRT or ST alone, not in combination with aerobic exercise, in women with PCOS (Table 4). In a non-randomized, case–control study involving a four-month intervention with PRT for one hour three days per week with linear periodization, effects on the sex hormones were mixed. T and FAI were lowered, but A4 and prolactin increased and SHBG decreased; no changes were observed in E2, LH, FSH, FI, or HOMA-IR [126,127,128]. A similar 16-week study of PRT with linear periodization also found lower T, but elevated A4 post-intervention [129]. In the previously discussed RCT involving a 10-week intervention of HIIT, a third arm with ST had lower FAI, higher SHBG, and lower AMH post-intervention, with no changes in T, APN, or leptin [116].

Limitations of these studies include selection bias for non-randomized trials. The lack of studies in this area, in combination with the promising effects of PRT on T, highlight the need for further investigation on the effectiveness of this type of resistance training on lowering androgen levels. Together with data from studies using only aerobic exercise and those using aerobic exercise and PRT or ST, these findings suggest that resistance training may help improve androgen levels, which may be complemented by vigorous aerobic activity.

### 3.4. Yoga

As with PRT and ST, few studies have examined the effectiveness of yoga in improving hormones in women with PCOS (Table 5). Yoga involves controlled breathing exercises and holding positions, often in a specific sequence and for a set amount of time. In an RCT comparing yoga to conventional exercise in adolescent girls with PCOS, a 12-week supervised intervention included 90 one-hour sessions that included surya namaskara (sun salutation), asanas (postures), pranayama breathing exercises, relaxation, and meditation in the yoga group. The conventional exercise group included walking, standing, sitting, and supine exercises with no yogic concepts or breathing exercises. Participants in the yoga group had lower TT, LH, FI, HOMA-IR, and AMH post-intervention [130]. In an RCT with adult women with PCOS, the three-month intervention included a guided yoga class for one hour three days a week, with pranayama breathing exercises, restorative asanas, vinyasa flows, mindfulness, and meditation. Participants in the yoga group had lower fT that persisted three months after the conclusion of the intervention, lower APN, and DHEA that trended lower; A4, DHEA-S, FI, and HOMA-IR did not change [131].

Limitations of these studies include small sample sizes, participant noncompliance with the intervention, and high attrition rates. Further studies are warranted to confirm these findings, to examine which aspects of yoga practice provide the most benefit for hormonal improvement in PCOS, and to investigate the frequency and duration of yoga practice that needs to be maintained to result in lower androgen and insulin levels.

### 3.5. Single Bout Exercise

While the majority of studies involving exercise in women with PCOS have utilized an intervention over several weeks or months, a couple have looked at the effects of a single bout of exercise on parameters associated with PCOS. Of these, one that has examined insulin and proteins in the insulin signaling pathway used a case–control study design with 40 min of walking at the participant’s target exercise heart rate. This single bout of exercise resulted in a decrease in serum insulin, even more so for women with PCOS than the non-PCOS controls [132].

The sex hormones are steroid hormones that may rise or fall over longer periods of time than can be detected after a single bout of exercise. However, peptide hormones fluctuate more rapidly and would be worth further examination. Additional studies are warranted on insulin levels following single bout exercise in women with PCOS as compared to women without the disorder, as well as studies on APN, leptin, and other peptide hormones.

## 4. Discussion

### 4.1. Impact of Exercise on Hormones Related to PCOS Pathology

#### 4.1.1. Sex Hormones

Exercise interventions with aerobic physical activity largely did not result in changes in the sex hormones, with a few exceptions. Two studies with a 16-week intervention involving brisk walking or jogging at least three days per week resulted in lower fT, DHEA-S, E1-S, and E2 [95] in the study with a weekly participant follow-up, and lower TT and FAI in the study with personal trainer supervision and a HRM for targeting intensity [90,91,93]. A 24-week cycling intervention with medical personnel supervision and continuous electrocardio monitoring resulted in improved T, FAI, and SHBG levels in ovulatory participants at 12 and 24 weeks, and study participants also reported increased intensity of their leisure time physical activity [96]. No changes were observed in A4 [84,86,88,92,101,133] or P [84,86,87,88,101,110,111,133] in any studies.

Combinations of aerobic exercise and PRT improved T and SHBG in both exercise plus diet arms of an RCT at 10 and 20 weeks, whereas FAI improved in all groups (including the diet-only group) [120]. HIIT improved DHEA-S but not T in a 10-week program with walking/jogging and/or cycling [116], whereas a 12-week program with aquatic HIIT improved fT, TT, FAI, and SHBG in addition to DHEA-S [118].

Of the limited studies examining PRT interventions exclusively, both found improvements in T with elevated A4 [126,127,128,129]. In one of the studies, FAI and SHBG also improved, PRL increased, but there was no change in E2 [126,127,128].

There have only been two reported studies on yoga intervention in adolescents or women with PCOS to date. In each, an improvement in TT [130] or fT was observed [132], and DHEA trended lower, although DHEA-S and A4 did not change in a study with adult women [131].

Together, the current evidence suggests that PRT and yoga may promote improvements in T. This suggests that exercises that promote muscle growth, particularly of glycolytic, fast-twitch muscle fibers, may result in the utilization of T and lower levels in the blood. This and other mechanisms may be responsible for these improvements and warrant further investigation. Aerobic exercise is unlikely to impact androgens, although closely monitored exercise and/or long-term incorporation of aerobic exercise may provide some benefit. The improvements in multiple androgens with 12 weeks of aquatic HIIT are intriguing and warrant further investigation.

#### 4.1.2. Insulin

Evidence from RCTs and case–control or single-arm studies with exercise intervention protocols that involved aerobic exercise at a specified VO_2_max collectively showed improvements in FI [84,86,88,93,101], AUC_ins_ [84,86,133], or other measures of insulin responsiveness such as GIR [87,88], IS [87,108], HOMA-IR [88,101], and glucose uptake or GDR [107,108]. Protocols in case–control studies that used intensified exercise also resulted in greater GIR and lower FI [103,104,105] and HOMA-IR [103,105].

In RCTs involving aerobic exercise in combination with PRT yielded improvements in FI [119], HOMA-IR [118], or FI and HOMA-IR [120,123], although the improvement in FI and HOMA-IR was seen in both aerobic exercise only or aerobic exercise and resistance training [120], suggesting that the improvement is a result of the aerobic exercise intervention. This is further supported by the findings that HIIT, but not ST, improved FI and HOMA-IR in an RCT with three parallel arms [116]. The comparison of HIIT versus MICT in one study showed improved FI only in the HIIT group, suggesting that the intensity of aerobic exercise may also play a role.

The two studies examining the effects of PRT on women with PCOS did not find changes in FI or HOMA-IR [126,127,128], or did not investigate metabolic parameters [129].

In the RCT with yoga intervention in adolescent girls with PCOS, FI and HOMA-IR improved [130], although this was not the case in an RCT with adult women with PCOS [131]. The average BMI in the adult group was greater than that in the adolescents, and women with PCOS may have already had established metabolic dysfunction, which could potentially explain this difference.

Together, aerobic exercise, particularly that maintained at a target VO_2_max or more vigorous intensity, appears to improve measures of insulin responsiveness and sensitivity. PRT or strength training alone does not appear to improve these measures. These findings suggest that the improved insulin responsiveness may come with habitual increased fuel demand by the muscles during aerobic exercise; however, studies are needed to investigate the underlying mechanisms of improved insulin responsiveness with physical activity. Yoga may improve insulin measures in younger and/or leaner patients with PCOS, although further studies are needed to elucidate the effectiveness in women of varying ages, BMI, and metabolic health.

#### 4.1.3. LH and FSH

Aerobic exercise interventions generally did not alter the LH or FSH levels in women with PCOS [84,86,88,92,97,100,101,110,111,133]. Combination exercise largely did not alter LH and FSH [119,121,122,123], with the exception of one study using aquatic HIIT that resulted in reduced LH and increased FSH [118]. The case–control study of PRT in women with PCOS that examined LH and FSH did not find any change in these hormones [126,127,128]. The yoga study that investigated LH and FSH in adolescent girls with PCOS did find a decrease in LH and no change in FSH [130]. Overall, the exercise interventions studied to date do not appear to consistently alter LH or FSH.

#### 4.1.4. Adipokines

Of the two studies that examined APN after an aerobic exercise intervention, neither resulted in a change in total APN [98,109]. One study also examined HMW APN and leptin, and found no difference in HMW APN, but did find a decrease in the ratio of leptin to HMW APN, and a trend toward lower leptin [109]. An RCT examining 10 weeks of HIIT or ST did not find any changes in the total APN levels [116], although another study did find increased APN following 12 weeks of HIIT [117]. Adipokines were not examined in the studies with PRT interventions [126,127,128], or in the study of yoga intervention in adolescents with PCOS [130]. APN decreased in women with PCOS following a three-month yoga intervention [131].

Additional and larger studies are needed in order to better assess the impact of aerobic or combination exercise, PRT or ST, and yoga on adipokines in women with PCOS. In addition to APN and leptin, other adipokines such as resistin and visfatin should be investigated following various forms of exercise interventions. In addition, as these hormones are produced by adipose tissue, correlations between serum levels of adipokines and adiposity or BMI would be appropriate.

#### 4.1.5. AMH

Of the two studies that examined AMH levels after an aerobic exercise intervention in women with PCOS, the 12-week protocol with intensified aerobic exercise for 1 h three days per week improved the AMH levels in a small sample [102]. A larger cohort following a 16-week protocol with brisk walking for 30–45 min at least three days per week did not have altered AMH levels [99]. An RCT that examined combined exercise did not find any changes in AMH [121,122], although an RCT comparing HIIT and ST did find lower AMH in the ST group [116]. Studies involving only a PRT intervention did not examine AMH levels [126,127,128]. The one yoga study that investigated AMH levels found decreases in both the yoga and conventional exercise groups [130].

Few studies have examined AMH following an exercise intervention. Although this is more a biomarker for PCOS severity and proxy for ovarian reserve in women [63,65,134], it would be interesting to investigate the impact of longer-term exercise interventions on AMH levels, as this may provide some insight into improvements in fertility and ovarian function.

### 4.2. Physical Activity Habits in Adolescents and Women with PCOS

Though not part of this systematic review, several groups have investigated the lifestyle habits of adolescent girls and women with PCOS. Some surveys of physical activity self-reported by women with PCOS indicate that frequency and total time spent on exercise are comparable to that of women without the disorder [135,136], including comparable metabolic equivalent of tasks (METs), exercise intensity, and use of exercise for weight management [137]. A correlative investigation of the number of steps per day suggests that women with PCOS who take more steps in a day may have improved FAI and HOMA-IR [136]. In addition, women with PCOS who exercise more vigorously had better metabolic profiles than those with less intense exercise, including lower FI and HOMA-IR, as well as higher SHBG [138].

A study of adolescent girls with or without PCOS found that those with the disorder were less physically active, including less frequent and lower intensity physical activity [139]. In another study, self-reported habits indicated that obese women with PCOS spent less time on moderate-intensity physical activity in a seven-day period compared with normal weight women with PCOS [140]. In a survey addressing barriers to physical activity, women with PCOS were more likely to report a lack of confidence in maintaining physical activity and fear of injury compared with women without PCOS, and more women with PCOS reported a desire to control a medical condition as a motivation for exercise [141].

Taken together, these findings suggest that adolescent girls with PCOS and women with both obesity and PCOS may be less likely to exercise and may exercise less frequently and with lower intensity when they do. These lower activity levels are likely insufficient to deliver metabolic benefits with respect to FI and HOMA-IR, as well as the potential benefits on androgen levels. Adolescents and patients with obesity should be screened for PCOS, and counseled to incorporate exercise activities that are accessible and enjoyable for them to increase their physical activity. Counseling should also include a discussion of the barriers to physical activity, and the importance of exercise intensity in improving hormone and metabolic health.

### 4.3. Limitations and Need for Additional Studies

While most studies of exercise intervention in women with PCOS have focused on aerobic exercise, few have examined PRT or ST alone; current evidence suggests a potential benefit in improving androgen levels with the latter and deserves further investigation. Studies of HIIT and yoga have also yielded promising benefits with respect to androgens and insulin sensitivity, although larger and additional studies are required to confirm these findings.

RCTs that include a no intervention control group and exercise intervention group(s) are the gold standard for clinical investigations and are necessary to determine the impact of physical exercise on hormones related to PCOS presentation and pathology. Given the particular importance of androgens and insulin in PCOS pathogenesis, future studies should focus on these as primary outcomes. Larger studies, particularly those which allow subgroup analysis to determine the impact of age, BMI, and weight loss, are needed in order to better understand the impact of exercise on individuals and allow for more specific guidelines for patients of differing demographics. Attrition rates tend to be high in long-term intervention studies such as yoga [142], so careful power analysis, recruitment, and retention will be needed in order to conduct such studies and complete a meaningful analysis of the data.

## 5. Conclusions

### 5.1. Recommendations for Guidelines

Considering the current body of evidence on the impact of various types of physical exercise on hormone health in women with PCOS, the following are recommended guidelines for healthcare providers and patients with PCOS:

1. All patients should be asked about current physical activity type(s), frequency, duration, and intensity. Adolescents and obese patients in particular should be asked about barriers to physical activity and counseled appropriately;
2. Vigorous aerobic activity at least three days per week for 30 min or more is recommended. A heart rate monitor or VO_2_max guided intensity levels (≥60% VO_2_max) are advisable to obtain insulin-related benefits;
3. Combination exercise that includes PRT or ST three days per week on non-consecutive days may provide additional benefits with respect to androgen levels;
4. Yoga may be a desirable activity to include as a regular physical exercise, although further studies are warranted to confirm its benefits with respect to androgens and insulin responsiveness.

### 5.2. Overall Conclusions

Physical exercise is part of the lifestyle recommendations for women with PCOS. Vigorous aerobic exercise, especially when practiced consistently over the long-term and in conjunction with heart rate and/or VO_2_max monitoring, can improve measures of insulin sensitivity. Improvements in androgens are more likely with resistance or strength training, although further studies are warranted for confirmation. The impact of yoga on insulin sensitivity and androgens appears promising and requires further study. In addition, further investigations are needed to determine the effects of different forms of exercise on adipokines and AMH in women with PCOS.

## Figures and Tables

**Table 1:** Criteria for the diagnosis of PCOS.

1990 National Institutes of Health (NIH) Criteria	Rotterdam Criteria	Androgen Excess Criteria
Demonstrates both: Clinical and/or biochemical hyperandrogenism Oligo- or anovulation	Demonstrates at least two of three: Clinical and/or biochemical hyperandrogenism Oligo- or anovulation Polycystic ovaries	Demonstrates: Hirsutism and/or hyperandrogenemia Also demonstrates at least one: Oligo- or anovulation Polycystic ovaries
Plus: Exclusion of other causes of androgen excess and anovulatory infertility	Plus: Exclusion of other causes of androgen excess and anovulatory infertility	Plus: Exclusion of other causes of androgen excess and anovulatory infertility

Exclusion of thyroid disease, disorders of the adrenal glands (including nonclassical congenital adrenal hyperplasia), and hyperprolactinemia.

**Table 3:** Studies of high intensity interval training and combination exercise interventions on hormones in women with PCOS.

Study	Participants	Exercise Intervention	Hormonal Changes	Unchanged
Prospective, intensified aerobic ex., case-control [115] Trial ISRCTN84763265	20 PCOS, 14 non-PCOS; 20–40 y, BMI > 27; NIH criteria	12-week intensified aerobic ex; 3 h/wk; added 8-week HIIT on treadmill, mod. intensity: 70% VO_2_max or 75–85% HR_max_, HIIT: 6x 5 min intervals 95–100% VO_2_max or HR_max_, 2 min rest, up to 8 reps and rest down to 1 min by 8 wk; HRM	↑GIR	PEDF
RCT w/3 parallel arms (HIIT, ST, no ex) [116] Trial NCT01919281	31 PCOS (~10/group); 27.2 ± 5.5 y; BMI 26.7 ± 6; Rotterdam criteria	10-week HIIT or ST; 3 d/wk; HIIT: 2 d/wk of 4 × 4 min at 90–95% HR_max_ w/ 3 min mod. II at 70% HR_max_, plus 1 d/wk 10× 1 min at max intensity w/1 min rest/low II; walking/running and/or cycling; at least 1 d/wk supervised; HRM; ST: 8 dynamic drills at 75% resistance of 1 RM, 3× 10 reps w/1 min rests	↓FI, HOMA-IR (HIIT) ↓DHEA-S (HIIT) ↓FAI, AMH (ST) ↑SHBG (ST)	T, APN, Leptin
Nonrandomized, HIIT or MICT [117]	20 PCOS (10/group); mean age 25 y; BMI 21.2–41.6; Rotterdam criteria	12-week HIIT or MICT program; 3 d/wk for 30 min; HIIT: 2 min run, 2 min walk; MICT: run at mod. tempo, constant speed	↓FI (HIIT) ↑APN (HIIT) *Trended in MICT*	Leptin, vaspin
RCT w/2 arms (aquatic HIIT+met, met) [118]	30 PCOS (15/group); 20–35 y; BMI ≥ 30; Rotterdam criteria	12-week aquatic HIIT; 3 d/wk for 30 min with 4 × 4min intervals, each with 8 rounds of 20 s max II w/10 s rest, and 1 min rest between each 4 min bout; HRM	↓LH, ↑FSH ↑SHBG ↓fT, TT, FAI, DHEA-S ↓HOMA-IR	N/A (hormones)
RCT w/2 arms (diet or diet+ex) [119]	5 PCOS (diet), 7 PCOS (diet+ex); mean BMI 36.6; Rotterdam criteria	12-week aerobic and resistance ex; 3 d/wk for 30 min at 70–85% HR_max_ plus 12 resistance ex of 2 × 10 reps progressing to 3 × 15 reps, increasing wt 5%; total of 90 min sessions; supervised	↓FI (both groups)	LH:FSH T, FAI, SHBG
RCT w/3 parallel arms (diet, diet and aerobic ex., diet and aerobic-resistance ex) [120]	94 PCOS (~30/group); 29.3 ± 0.7 y; BMI 36.1 ± 0.5; Rotterdam criteria	20-week walking/jogging program 5 d/wk or walking/jogging 3 d/wk plus PRT 2 d/wk on nonconsecutive days;	↓FI, HOMA-IR (both ex. groups, 10 wk; all at 20 wk) ↓T (both ex. groups, 10, 20 wk) ↓FAI (all at 10, 20 wk) ↑SHBG (both ex. groups, 10, 20 wk)	N/A (hormones)
RCT w/3 arms (diet, ex, diet+ex) [121,122] Trial ISRCTN48342048	57 PCOS (19/group); 18–40 y; BMI > 27; Rotterdam criteria	4-month individualized moderate exercise (aerobic and strength training); moderate to high intensity; 2–3 d/wk; 45–60 min; physiotherapist supervised	N/A (hormones)	LH, FSH T, fT, SHBG AMH FI, HOMA-IR, IGF-1
RCT w/2 arms, ex or no ex [123]	32 PCOS (16/group); BMI < 25; Rotterdam criteria	8-week aerobic and resistance ex; 3 d/wk for 50–60 min; treadmill and step, resistance band; supervised by physiotherapist	↓FI, HOMA-IR	FSH, LH E2, TT, fT
RCT w/2 arms (ex or ex+met) [124]	66 PCOS; 24.4 ± 4.3 y; BMI 26 ± 4; Rotterdam criteria	6-month marching in place; 3 d/wk for 30 min; monitored by investigator for first 3 months to ensure HR ≥ 120 bpm	N/A (hormones)	T

Abbreviations: A4, androstenedione; Anov, anovulatory; APN, adiponectin; AUC, area under curve; cd, cycle days; ex, exercise; HIIT, high intensity interval training; HRmax, maximum heart rate; II, intensity interval; ins, insulin; ISI, insulin sensitivity index; met, metformin; MICT, medium intensity continuous training; NW, normal weight; OB, overweight or obese; OGTT, oral glucose tolerance test; ov, ovulatory; pbo, placebo; PA, physical activity; PEDF, pigment epithelium-derived factor; reps, repetitions; RM, repetition maximum; ST, strength training; wt, weight.

**Table 4:** Studies of resistance exercise interventions on hormones in women with PCOS.

Study	Participants	Exercise Intervention	Hormonal Changes	Unchanged
Nonrandomized, case-control [126,127,128] Brazilian Clinical Trials Registry (ReBec: RBR-7p23c3)	45 PCOS, 52 non-PCOS; 18–37 y; Rotterdam criteria	4-month PRT; 3 d/wk of PRT for 1h w/microcycles of increasing intensity, decreasing reps (linear periodization)	↑PRL ↑A4 ↓T, FAI ↓SHBG	FSH, LH E2 FI, HOMA-IR
Prospective, case-control, PRT [129]	43 PCOS, 51 non-PCOS; sedentary; 18–37 y; BMI 18–39.9; Rotterdam criteria	16-week PRT; 4 microcycles of 4 wk w/linear periodization; 10 ex w/10 reps/ex; supervised	↓T ↑A4	N/A (hormones)

Abbreviations: PRT, progressive resistance training.

**Table 5:** Studies of yoga interventions on hormones in women with PCOS.

Study	Participants	Exercise Intervention	Hormonal Changes	Unchanged
RCT w/2 arms (yoga, CE) [130] Central Trial Registry of India No.: REFCTRI-2008 000291	90 PCOS; 15-18 y; mean BMI 20.8; Rotterdam criteria	12-week supervised yoga or CE; 90 sessions total of 1 h; yoga: surya namaskara, asanas, pranayama, relaxation, meditation; CE: walking, standing, sitting, supine ex w/no yogic concepts or breathing	↓FI, HOMA-IR (yoga) ↓LH (yoga) ↓TT (yoga) ↓AMH (yoga, CE)	FSH, PRL
RCT w/3 arms (yoga, OMT, no ex) [131] Trial NCT03383484	22 PCOS (13 yoga, 9 no ex); 22–43 y; BMI 20–48; Rotterdam criteria	3-month supervised mindful yoga; 3 d/wk for 1 h; yoga: pranayama, vinyasa flows, restorative asanas, mindfulness, meditation; follow-up 3 mo post-study	↓fT (yoga, persisted 3 mo) ↓APN DHEA trended lower	DHEA-S, A4FI, HOMA-IR

Abbreviations: CE, conventional exercise; mo, month; OMT, osteopathic manipulative treatment.

## Abbreviations

A4	Androstenedione
AMH	Anti-Mullerian hormone
ANP	Atrial natriuretic peptice
APN	Adiponectin
AUC	Area under the curve
Anov	Anovulatory
BMI	Body mass index
bpm	Beats per minute
CAT	Continuous aerobic training
cd	Cycle days
CG	Control group
d	Days
DHEA-S	Dehydroepiandrosterone sulfate
E1-S	Estrone sulfate
E2	Estradiol
Ex	Exercise
ExEE	Exercise energy expenditure
FAI	Free androgen index
FBG	Fasting blood glucose
FFM	Fat free mass
FI	Fasting insulin
FSH	Follicle stimulating hormone
fT	Free testosterone
GDR	Glucose disposal rate
GIR	Glucose infusion rate
HIIT	High intensity interval training
HMW	High molecular weight
HOMA-IR	Homeostatic model assessment of insulin resistance
HR	Heart rate
HRM	Heart rate monitor
HRR	Heart rate reserve
IAT	Intermittent aerobic training
II	Intensity interval
Ins	Insulin
IR	Insulin resistance
IS	Insulin sensitivity
LH	Luteinizing hormone
Met	Metformin
MICT	Medium intensity continuous training
Mo	Month (s)
N/A	Not applicable
NW	Normoweight
OB	Overweight or obese
OC	Oral contraceptive
OGTT	Oral glucose tolerance test
Ov	Ovulatory
PA	Physical activity
PRL	Prolactin
PRT	Progressive resistance training
RCT	Randomized controlled trial
Reps	Repetitions
RM	Repetition maximum
SHBG	Sex hormone binding globulin
ST	Strength training
T	Testosterone
T4	Thyroxine
TSH	Thyroid stimulating hormone
TT	Total testosterone
Wk	Week (s)
Wt	Weight
Y	Year (s)
